# Hip width and metabolic energy expenditure of abductor muscles

**DOI:** 10.1371/journal.pone.0284450

**Published:** 2023-04-18

**Authors:** Patricia Ann Kramer, Adam D. Sylvester

**Affiliations:** 1 Department of Anthropology, University of Washington, Seattle, Washington, United States of America; 2 Center for Functional Anatomy and Evolution, The Johns Hopkins University School of Medicine, Baltimore, Maryland, United States of America; University of Cambridge, UNITED KINGDOM

## Abstract

Despite a paucity of physiological evidence, simplistic biomechanical analyses have led researchers to assume that humans who have wider hips use more energy to walk. Pitting biomechanical first principles against physiological data has led to little deepening of our understanding of bipedalism and its evolution. Both approaches, however, use proxies for the energy used by muscles. We decided to approach the question directly. Using a musculoskeletal model of the human body that estimates the metabolic energy expenditure of muscle activation for 48 people (23 women), 752 trials were evaluated. Metabolic energy consumption for the abductor muscles was summed over a stride to create total abductor energy expenditure. We calculated the maximum hip joint moment acting in the coronal plane and the functional distance between the hip joint centers. We hypothesize that wider hips would be correlated with both maximum coronal plane hip moment and increased total abductor energy expenditure when mass and velocity were controlled. Linear regressions with multiple independent variables, clustered by participant to control for the non-independence of the data points, were performed in Stata. We found that hip width does not predict total abductor energy expenditure, although mass and velocity combine to predict 61% of the variation (both p<0.001). Maximum hip joint coronal plane moment is predicted by pelvic width (p<0.001) and, in combination with mass and velocity (both p<0.001), explains 79% of the variation. Our results indicate that people use their morphology in ways that limit differences in energy expenditure. Consistent with recent discussion, intraspecific variation might not be useful to understand differences among species.

## Introduction

Many creatures use metabolic energy to move themselves through their environment in search of food, water, shelter, mates, and most other requirements of survival and reproduction, and individuals who use less energy on movement tasks presumably have more energy available to devote to other evolutionarily important tasks such as investment in more and/or higher quality offspring. Understanding how movement is influenced, or not, by form has received considerable attention in the evolutionary literature (e.g., avian flight [1]; sauropod body plans [2]; shark musculotendinous elements [3]). In hominins, this examination has frequently focused on examining the effect of the morphology of the pelvis (e.g., [4]) and/or lower limb (e.g., [5]) on the efficiency of bipedal locomotion.

Like other mammals [6], the energy that humans use to walk is linked to mass and the velocity of travel. These two variables explain much of the variation in energy expenditure among people [7], but other individual characteristics have been found to be related to energy expenditure less consistently. Of particular interest in the evolution of hominin locomotion has been the effect of pelvic width on the effectiveness of walking. In a pivotal Scientific American article [8], Washburn described an “obstetrical dilemma” (OD) that Rosenberg [9] refined: efficient bipedalism necessitates a narrow distance between the acetabula but birth of large-brained offspring requires a wide birth canal. This view has been widely held (although see [10]) and its implications actively researched and debated for 60 years [11, 12].

In considering the implications of pelvis width on locomotion, Lovejoy and colleagues [13] and Ruff [14] calculated joint reaction forces in the coronal plane of midstance using the center of the femoral head as the pivot point about which weight (mass times gravitational acceleration) and abductor muscle forces were balanced in a static freebody. Lovejoy and colleagues [13] focused on the impact of the morphology of Sts 14 on femoral head pressure, which they found to be similar to, or less than, that of modern humans when iliac blade flaring and femoral neck length were considered. Lovejoy and colleagues did consider the implications of pelvic width constraints on locomotion, but they concluded that total body width (e.g., iliac crest breadth) was constrained, forcing modern humans into a compromise that resulted in less flared (and hence less effective) iliac blades and large femoral heads (their Fig 13). Ruff [14] found that the impact of a wide pelvis (based on hip joint center) on femoral shaft loading could not be ameliorated by femoral neck length or iliac blade flare. Neither Ruff nor Lovejoy and colleagues were able, at the time, to evaluate the effect of muscle redundancy on bone and joint loading patterns, nor did they consider their static freebody diagrams in other planes or at other points in single limb support. They also did not consider the physiological energetic cost of producing muscle force, but rather concentrated on mechanical considerations (i.e., bone loading).

This early work on the impact of pelvic morphology on the force induced in the hip abductor muscles is connected to understanding the activation of those muscles during human walking. Electromyography (EMG) was instrumental in demonstrating the role of gluteus medius (a superficial hip abductor) in pelvic stabilization (e.g., [15–17]) and considerable effort has been expended in extending and expanding the understanding of muscle firing patterns in their timing within the gait cycle and their magnitude across conditions (e.g., [18–20]). While the importance of muscular activity to provide pelvic stabilization is well established, the role of pelvic morphology in abductor muscle activation has received less attention. No significant difference between males and females in gluteus medius activity has been established for walking (e.g., [20, 21]). Nonetheless, bitrochanteric breadth is associated with gluteus medius activity [22], indicating a role for pelvic morphology. The strength of this relationship and how muscle activation impacts muscle energy use remains unclear.

Other researchers (e.g., [10, 23]) have focused on searching for the impact of pelvic width on metabolic energy expenditure by measuring the volumetric rate of oxygen consumption of walking (VO_2_). These results have found no statistically significant relationship between hip width and energy expenditure (e.g., [10]), but this lack of support has been attributed to a paucity of data—both too few participants and too little variation among them [11]. One problem with determining the energetic requirements of walking is that physiological variables are complex and vary among people in ways that are not fully understood. Individuals cannot serve as their own control for these physiological variables in energetic work because pelvic width cannot be changed in living people. Additionally, assessment of VO_2_ requires that the individual reach a steady state of oxygen consumption (usually 3 or more minutes), limiting most energetic studies to treadmills (or, occasionally, athletic tracks) where steady state can be achieved, and all require equipment to measure oxygen, carbon dioxide, and air flow. Consequently, energetic studies frequently suffer from a limited number of trials and/or participants. Even more importantly, VO_2_ is a measure of whole-body oxygen consumption and necessarily includes the oxygen use of other organs and processes, many of which are not fully understood. Using resting metabolic rate as a covariate attempts to control for these other uses of oxygen but there is currently no way to measure the oxygen use of individual muscles. Additionally, and we think most importantly, selection may have shaped the range of variation in human pelvic width to preclude deleterious effects on locomotion such that “… small effects on locomotion efficiency … are compensated by other anatomical features or changes to gait kinematics (Wall-Scheffler and Myers (2017))” [11, 24].

“Hip width,” “pelvic breadth,” or “biacetabular breadth” is also a complex variable to evaluate empirically. Lovejoy and colleagues’ [13] and Ruff’s [14] calculation depended on the distance from the centerline of the body to the hip joint center of rotation, but the OD arises from a constraint on pelvic canal breadth/diameter. The pelvic canal diameter can be defined as a mediolateral measure (e.g., the maximum distance between the linea terminalis), but this dimension is not necessarily the smallest dimension in the true pelvis. Rather, the midplane distance between spinous processes is frequently the minimum diameter in a given individual [25]. Additionally, mediolateral pelvic breadth is frequently operationalized as biacetabular breadth. While presumably correlated, these values are not the same: the distance between the hip joint centers includes the soft tissue of the hip and the radius of the femoral head; the minimum pelvic canal breadth can be located in multiple locations (e.g., inlet, outlet, midplane); and biacetabular breadth may not coincide with the minimum pelvic canal diameter because the canal is not necessarily of circular cross section. Further, the location of the acetabulum and, hence, the orientation of femoral head is variably anteverted (i.e., located anterolaterally on the pelvis) in adults [26].

Complicating matters further, determining the hip joint center of rotation, canal breadth, or the biacetabular breadth in a living individual—from whom energetic values can be determined—requires invasive imaging (e.g., [10]) or the use of regression equations that link palpable landmarks (e.g., ASIS) to some measure of hip width [27, 28]. This imaging requirement further limits the number of participants in any study. While minimum pelvic canal breadth is the critical dimension for birth, the location of the hip joint centers relative to the center of mass of the body is the relevant dimension for biomechanics. These distinctions are critical and the locomotor efficiency argument of others (e.g., [13, 14]) stems from the distance between the center of rotation of the hip joint, a functional not anatomical dimension.

Warrener and colleagues [10] used magnetic resonance images of their participants to calculate “biacetabular width” (by which they meant the distance between the femoral head centers) and moment arms for the hip abductor muscles. They found no impact of biacetabular width on the mass-adjusted energy expenditure (VO_2_) of walking or running. They also found that females did not differ from males in biacetabular width although females were shorter and lighter. Their sample was, potentially, biased by a requirement that the individuals were recreational runners, which may have created a selection bias in the participants. Potentially of more importance, they only evaluated a single walking velocity (1.5 m/s), they did not consider the impact of muscle redundancy on muscle force production, and they only evaluated midstance. Wall-Scheffler and Myers [23] used bitrochanteric breadth—the distance between the external surface points superficial to greater trochanters of the femora—as their proxy of hip width and they, too, found no support for a negative impact on energy expenditure for wider hips. Bitrochanteric breadth includes biacetabular breadth, hip joint soft tissue, proximal femoral shape, and considerable soft tissue superficial to the trochanters, making its correlation with biacetabular breadth less clear. Nonetheless, gait mechanics can be adapted to accommodate variation in form to produce similar function. Specifically, wider hips allow for a longer stride length relative to leg length [29, 30] and are associated with increased stability, which necessitates less mediolateral force development [23]. Hence, while wide hips potentially increase hip coronal plane moment and abduction muscle forces (e.g., [14]), wide hips also create mechanisms that use less energy to move [23].

Consequently, although the mechanical first principles, when simplified to one anatomical plane and when all else is held constant, unequivocally demonstrate an effect of hip joint center distance on hip coronal plane moment, (presumably) muscle force production, and, hence, energy expenditure, the anatomical and physiological data of living people are inadequate to confirm (or negate) the central tenet of the OD regarding metabolic energy expenditure. That is, the assumption at the heart of the OD—that pelvic width is constrained by locomotor energy expenditure—is unsupported by convincing empirical evidence in modern humans. Sufficient questions remain to be answered, however, before the metabolic energy clause of the OD can be completely rejected [11] and other potential causes (e.g., pelvic floor integrity [31, 32], femoral neck fracture risk [33] emphasized.

Given the complexity of the musculoskeletal system and of the motions that it produces, we expect that the empirical energetic evidence and the biomechanics are not in conflict. Rather inadequate understanding of both creates the appearance of conflicting results. Consequently, we decided to approach this problem from a different perspective utilizing musculoskeletal modeling which allows us to connect biomechanics to metabolic energy expenditure of the individual muscles directly. Although derived from earlier biomechanical studies that used 2D static analyses, we utilize 3D inverse dynamics in a musculoskeletal model. We apportion muscle forces using a muscle redundancy algorithm that includes the effect of muscle strength and moment arm. Inherent in the inverse dynamics approach is the inclusion of segment translational and angular accelerations as well as externally applied forces. Additionally, inverse dynamics analyses solve for muscle forces (and other parameters) at multiple time points in a step.

In studies of locomotion, whole body oxygen consumption (as measured by indirect calorimetry) is simply a convenient proxy for the energy that is used by the muscles to move the body or, even more distantly connected, the energy used by particular muscles. Muscles use metabolic energy and the characterization of the relationship between muscle activation and metabolic energy use has also received considerable attention (e.g., [34, 35]). We wondered, therefore, if the metabolic energy consumption of abductor muscles could be calculated across a stride and then evaluated to determine if hip width was predictive of this portion of muscle metabolic energy use. Further, musculoskeletal modeling allows for the determination of joint centers of rotation from the movement patterns of individual segments [36, 37]. Typically, the segment motion patterns are determined from optical motion capture in biomechanical laboratories. Joint center positions determined from movement are functional; they reflect what the body does with its morphology, rather than simply the underlying anatomical shapes.

We utilized existing data of 48 people (23 women; 25 men) who walked at multiple velocities [38], and we determined the metabolic energy use of individual muscle elements across a stride and the functional hip joint center distance of each individual. We ask if functional hip joint center distance is predictive of the metabolic energy expenditure of the hip abductor muscles when body mass and velocity of locomotion are controlled. We predicted that the functional distance between the hip joint centers would be a significant predictor of:

1. Maximum hip coronal plane moment and
2. The metabolic energy consumption of the hip abductor muscles in a stride.

## Methods

We utilized the existing data provided by Schreiber and Moissenet [38] which includes motion capture of 52 optical markers and ground reaction forces of 50 participants. The data of two participants were discarded due to marker irregularities, leaving 48 participants for this study (Table 1). Each participant’s data included 3–5 trials in each of five velocity categories: very slow (C1, <0.4 m/s), slow (C2, 0.4–0.8 m/s), slow normal (C3, 0.8–1.2 m/s), self-selected spontaneous (C4), and self-selected fast (C5). We decided that C1 trials were too slow (<0.5 m/s) to represent habitual walking in uninjured adults and so do not include those trials in this analysis, leaving 856 trials available for analysis. Additionally, trials which did not include both left and right feet, each in contact with a single force plate, were discarded.

**Table 1:** Mean and range of sample characteristics.

	Men (n = 25)		Women (n = 23)	
	Mean (SD)	Range	Mean (SD)	Range
Mass (kg)	78.2 (11.5)	50.8–98.0	63.2 (7.3)	50.0–80.5
Stature (m)	1.80 (0.066)	1.66–1.92	1.67 (0.054)	1.55–1.76
Leg length (m)	0.85 (0.041)	0.73–0.92	0.79 (0.041)	0.71–0.85
Hip joint center distance (m)	0.188 (0.016)	0.165–0.231	0.173 (0.013)	0.144–0.194
Bitrochanteric breadth (m)	0.372 (0.020)	0.332–0.412	0.380 (0.020)	0.349–0.431
	(n = 395 trials*)		(n = 357 trials*)	
Horizontal velocity (m/s)	1.04 (0.33)	0.359–1.96	1.11 (0.36)	0.482–1.99
Maximum Moment (Nm)	71.9 (15.1)	41–114	57.9 (12.7)	28–100
Pmet (Nm/s)	6022 (1759)	2424–11991	6187 (2161)	2321–16628

* All trials with one foot on one force plate and velocity < 2 m/s

**Fig 1:**
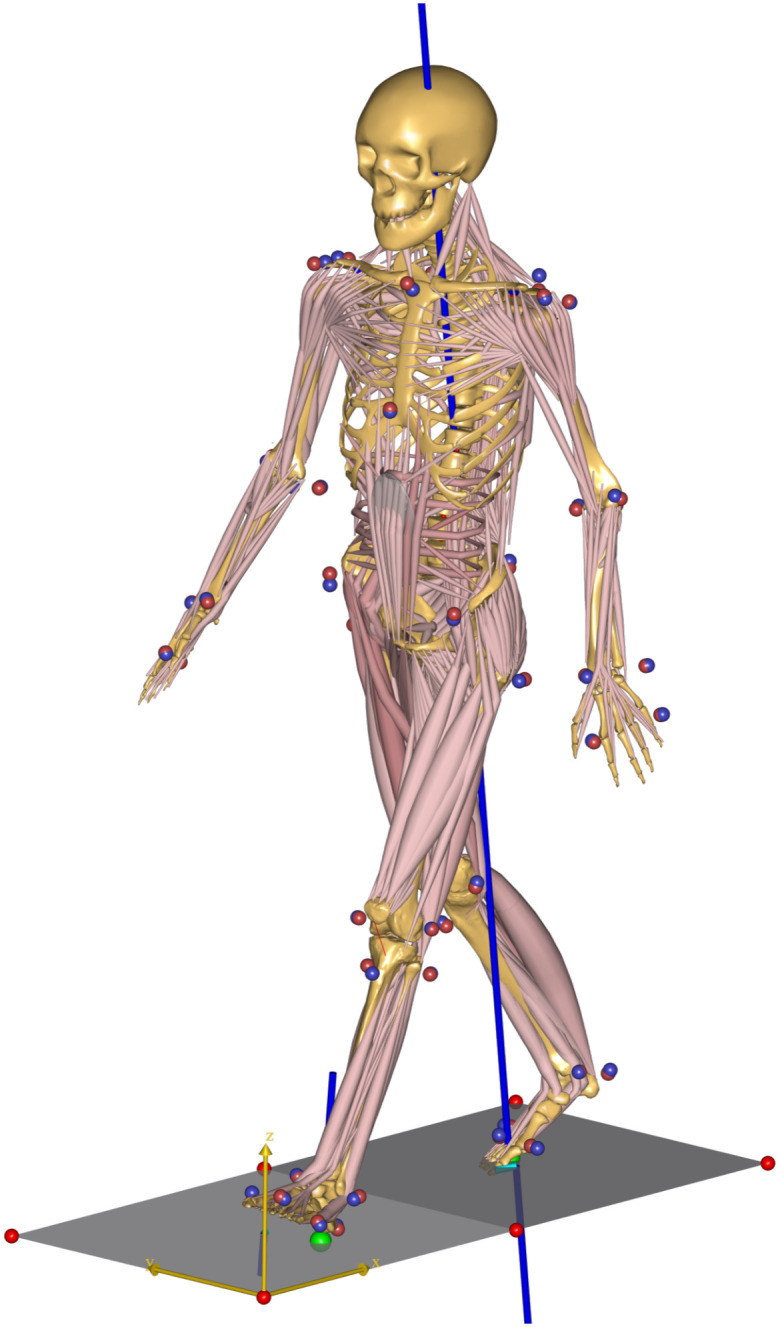
Musculoskeletal model of a walking human. The image captures a frame with both feet in contact with a (different) force plate (i.e., double stance). Muscle elements are shown in pink with skeletal elements in yellow-brown. Some muscles can be adequately represented with a single muscles element (e.g., sartorius) but others (e.g., gluteus medius) require many muscle elements. The location of experimental markers and marker drivers is indicated with red and blue spheres. The force plates are shown in grey and the ground reaction forces in blue, with the length of the line indicative of the magnitude of the force.

We created a musculoskeletal model in AnyBody (v7.3, AnyBody Technology, Denmark), starting from the ADL Gait (beta) Fullbody MoCap model (AnyBody Managed Model Repository AMMR v2.4.2), using the standard workflow described previously [24, 39, 40]. The MoCap model contains segments that represent the left and right upper limbs, trunk, and left and right lower limbs. Optical markers from the motion capture data of Schreiber and Moissenet (2019) were used to drive the full motion of all segments. The following segments comprise the lower limbs: pelvis, thigh, patella, shank, talus, and foot segments. The joints of the lower limb allow six total degrees of freedom (three rotations at the hip and one each at the knee (flexion/extension), ankle (plantarflexion/ dorsiflexion), and subtalar (inversion/eversion) joints). Forty-one lower limb muscles are composed from 169 muscle elements in each model lower limb. Muscle origins, insertions, and intermediate points as well as other muscle parameters were developed for the Fullbody MoCap Model from cadaveric data (Fig 1). To briefly recount the process, we determined the segment parameters (e.g., pelvis width, femoral length) using the first C4 trial (C4-01) that was analyzed using the Parameter Identification routine in AnyBody. This process determines the best fit set of marker driver positions and segment parameters (e.g., length, width) that produce the overall motion. Bilateral symmetry was imposed. With these data, we used AnyBody to solve the inverse dynamics and muscle redundancy problems. The result of these calculations include the force in each individual muscle element as well joint reaction forces and moments and positional information. The ADL Gait model uses the Margaria [34] equations to approximate muscle metabolic energy use: muscle metabolic power = muscle power/e, where e = 0.25 for concentric contractions and e = -1.25 for eccentric contraction. Muscle power is calculated from muscle force and velocity. Joint moments and metabolic energy expenditure (designated Pmet in AnyBody; in Nm/s) of each muscle element for each frame of data, along with pelvic width, were the principal variables of interest produced by the AnyBody analysis.

Custom written Matlab (Mathworks, Boston, MA) programs were used to extract the joint moment and Pmet data from the AnyBody results file. Gait events (heelstrike and toe off of each step), as determined by Scheiber and Moissenet [38], were used to establish the midstance (the middle frame between heelstrike and toe off) of each step on the two force plates that were available. Midstance of force plate 1 to midstance of force plate 2 constitutes a step that was used for all subsequent analyses. We then interpolated the moment and Pmet data to create 1% increments of this step in order to be able to compare among trials. Maximum hip coronal plane moments and the average mediolateral distances from the center of mass of the body to the hip joint center for the stance limb (either left or right depending on the step sequence of the trial) were determined. This moment acts to prevent the pelvis from dropping on the unsupported side of the body and is the analog of the hip moment as calculated for single limb stance in previous static analyses (e.g., [10, 13, 14]). The Pmet values for each frame in the step were summed for the muscle elements of gluteus medius and minimus. This summed Pmet represents the metabolic energy used by the activation of the muscle elements in the step to prevent pelvic drop. Including left and right muscles provides an estimate of a complete stride (i.e., a left and a right step). Additionally, we calculated horizontal velocity from the movement of the optical marker placed superficial to the tip of the spinous process of the seventh cervical vertebra (C7) and bitrochanteric breadth from the position of the optical markers placed over the left and right greater trochanters of the femur (designated FTC in the original data).

Linear regressions with multiple independent variables, clustered by participant for to control for the non-independence of the data points, were performed in Stata (StataCorp, College Station, TX) with statistical significance set at p ≤ 0.01. Dependent variables were maximum hip moment in the coronal plane and summed Pmet. Independent variables for each participant include mass (kg), stature (m), gender (men/women, as designated by Schreiber and Moissenet [38]), leg length (m), the distance between hip joint centers (m), and bitrochanteric breadth (m). Velocity was included as an independent variable for each trial. The potential for overlapping relationships among independent variables (multicollinearity) required iterative construction of regression models (i.e., stepwise regression) and examination of the added variable plots.

## Results

Of the 856 trials available, 104 of them had a problem with the input data (e.g., foot placement off the force plate) and were not included in the statistical analyses. These trials were spread throughout the participants; every participant had at least 12 trials included in the analyses.

**Fig 2:**
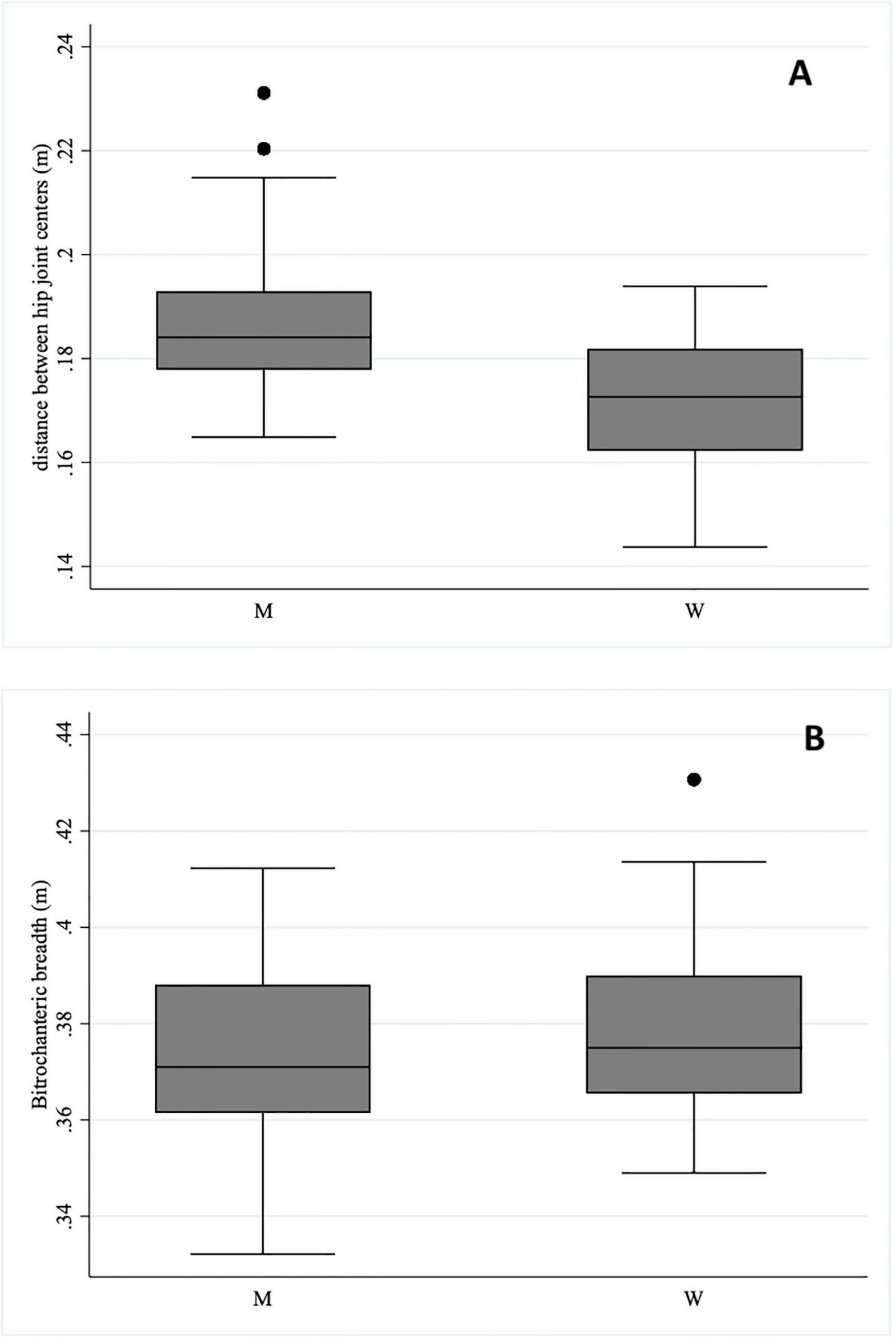
Functional distance between hip joint centers and bitrochanteric breadth in men (M) and women (W).

Before constructing the regression models, we evaluated the relationships among our independent variables. Men weigh more, are taller, and have longer legs than women (all p<0.001). Men also are wider than women between the functional hip joint centers (p = 0.001), but the distance between the trochanteric markers is not significantly different (Fig 2; Table 2). Stature predicts 22% of the variation among participants in the distance between the functional hip joint centers (p<0.001, r^2^ = 0.22) and gender does not improve the predictive ability. Mass predicts 53% of the variation in distance between the functional hip joint centers (p<0.001, r^2^ = 0.53) and gender does not improve the predictive ability. Stature predicts bitrochanteric breadth only when gender is included in the model (both p = 0.002, r^2^ = 0.19), while mass with gender predicts 67% (both p<0.001, r^2^ = 0.67). Distance between the hip joint centers explains 77% of the variation among individuals in the average mediolateral distance from the center of mass of the body to the hip joint center of the stance leg (p<0.001, r^2^ = 0.77 for both legs) and neither mass, stature, leg length, nor gender improve the predictive ability.

**Table 2:** Individual anthropometric characteristics. Mass and stature are from Schreiber and Moissenet (2019); hip joint center distance and bitrochanteric breadth calculated in the current analysis.

subject	Mass (kg)	Stature (m)	Hip joint center difference (m)	Bitrochanteric breadth (m)
SM2014001	67	1.66	0.176	0.354
SM2014002	65.4	1.64	0.180	0.382
SM2014003	50	1.56	0.144	0.349
SM2014004	72.5	1.77	0.173	0.361
SM2014005	73.5	1.83	0.179	0.353
SM2014006	73	1.76	0.177	0.364
SM2014008	57.1	1.66	0.175	0.370
SM2014009	86	1.88	0.208	0.396
SM2014011	63.4	1.8	0.171	0.349
SM2014013	61.3	1.7	0.158	0.374
SM2014014	92	1.8	0.178	0.382
SM2014015	67	1.58	0.187	0.374
SM2014016	76	1.69	0.194	0.414
SM2014019	73.8	1.76	0.171	0.403
SM2014022	59.8	1.71	0.182	0.370
SM2014024	87.5	1.92	0.181	0.388
SM2014025	80.5	1.66	0.192	0.431
SM2014029	89.9	1.89	0.186	0.381
SM2014030	60.7	1.7	0.177	0.359
SM2014031	67.2	1.77	0.165	0.371
SM2014033	63.5	1.6	0.180	0.375
SM2014034	89.6	1.84	0.190	0.375
SM2014040	56.5	1.55	0.159	0.358
SM2014046	61.8	1.65	0.188	0.366
SM2014048	61.5	1.64	0.171	0.379
SM2014049	72.2	1.74	0.184	0.366
SM2014050	61.9	1.64	0.157	0.390
SM2014051	88	1.91	0.191	0.388
SM2014052	79.5	1.82	0.179	0.380
SM2014053	62.8	1.72	0.166	0.389
SM2015002	74	1.74	0.192	0.377
SM2015003	87.2	1.77	0.215	0.411
SM2015004	62	1.7	0.171	0.362
SM2015007	60.2	1.66	0.176	0.365
SM2015013	73	1.69	0.162	0.404
SM2015015	68	1.73	0.183	0.409
SM2015017	60.5	1.67	0.173	0.386
SM2015020	95	1.79	0.220	0.393
SM2015021	58	1.69	0.165	0.375
SM2015026	51.5	1.71	0.161	0.350
SM2015027	65.5	1.72	0.182	0.332
SM2015030	86	1.87	0.192	0.370
SM2015032	50.8	1.72	0.173	0.339
SM2015035	81.5	1.77	0.193	0.370
SM2015037	66.1	1.76	0.195	0.371
SM2015041	74.6	1.88	0.194	0.378
SM2015042	98	1.83	0.231	0.412
SM2015043	74	1.78	0.178	0.346

**Fig 3:**
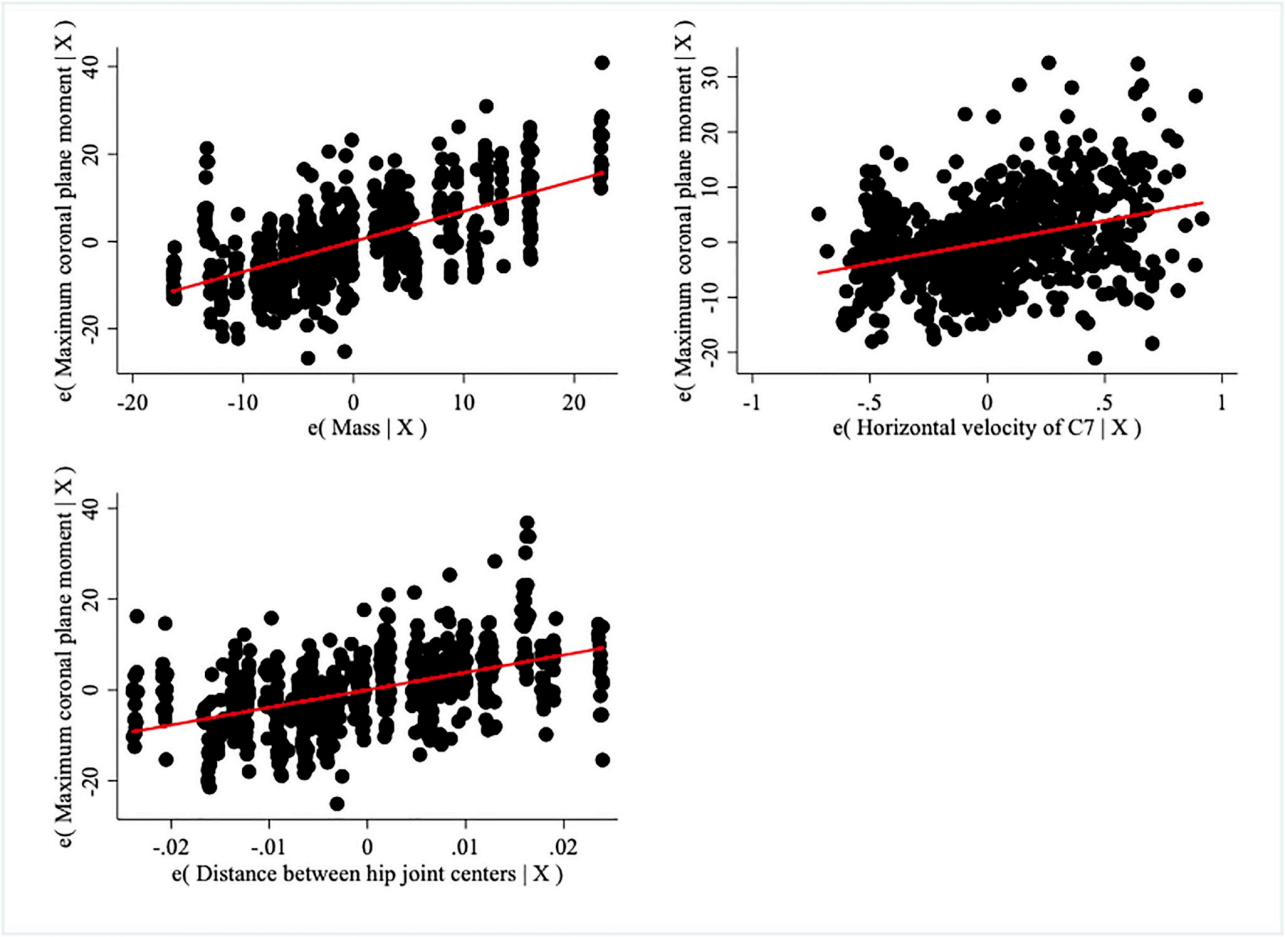
Contribution of mass, horizontal velocity of C7, and functional distance between hip joint centers to the prediction of maximum hip coronal plane moment when the effect of other variables is controlled. Sometimes called added variable or partial regression (leverage) plots, each panel demonstrates the effect of one independent variable (either mass, velocity, or distance) on the dependent variable when the effects of the other two independent variables are accounted for.

Over 70% of the variation in maximum hip coronal plane moment is explained by mass and velocity (both p<0.001, R^2^ = 0.71) and the distance between the hip joint centers improves the predictive ability by 8% (all p<0.001, R^2^ = 0.79) (Fig 3). Inclusion of stature, leg length, or bitrochanteric breadth does not improve the predictive ability of the model.

**Fig 4:**
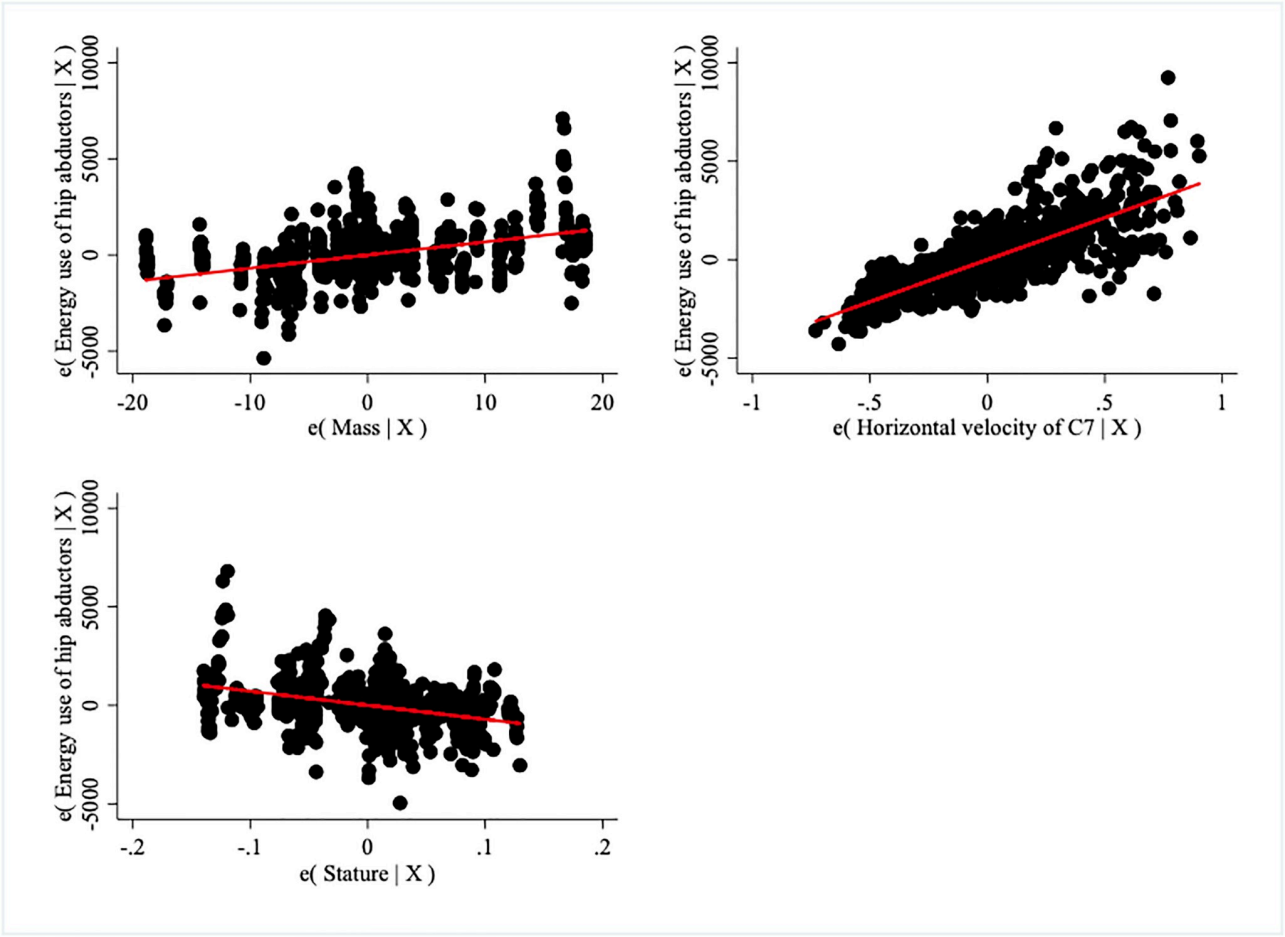
Contribution of mass, horizontal velocity of C7, and stature to the prediction of muscle metabolic energy expenditure of the hip abductors in a stride when the effect of other variables is controlled. Sometimes called added variable or partial regression (leverage) plots, each panel demonstrates the effect of one independent variable (either mass, velocity, or stature) on the dependent variable when the effects of the other two independent variables are accounted for.

Summed Pmet is predicted by mass and velocity (both p< 0.001, R^2^ = 0.61) and stature improves the predictive ability of the model (p = 0.004, R^2^ = 0.65) (Fig 4). Neither the distance between the hip joint centers nor bitrochanteric breadth improves the predictive ability.

## Discussion

Increasing distance between the hip joint centers increases the maximum hip coronal plane moment in a stride, in accordance with previous static analyses (e.g., [10, 13, 14]). Increasing distance between the hip joint centers does not, however, improve the estimation of the metabolic energy consumption of the abductor muscles, unlike the assumption of the OD but in agreement with empirical analysis (e.g., [10, 23]). Also, although men are absolutely wider (i.e., the distance between their functional hip joint centers is larger) than women, this difference appears to depend on differences in stature between the gender categories. The OD depends on the distance between the hip joints varying with the diameter of the true pelvis, believed to be wider in females (e.g., [25]). We cannot know the diameter of the true pelvis in this sample, but our results suggest that this assumption of the OD should be more rigorously evaluated.

That maximum coronal plane hip moment increases with increasing distance between the functional hip joint centers was expected because the moment is the direct result of the dynamic force exerted (a product of mass and acceleration) and the perpendicular distance between the line of action of that force and the hip joint center. Wider hip joints increase this distance. Beyond simple statistical significance, though, the hip joint center distance improves the predictive ability of the model by 8%, which could be of practical importance since this effect is experienced with every step. Nonetheless, greater than 20% of the maximum coronal plane hip moment is not explained by the morphological variables that we included and that would be available in more simplistic analysis approaches. This unexplained variation points to the complexity of movement and the requirement of using musculoskeletal models to assess function. If hip coronal plane moment was the sole determining factor in hip abductor muscle activation, then muscle energy use might be an important constraint on pelvic breadth among modern humans. But, muscle activation across a stride is influenced by the totality of movement (i.e., not a static snapshot of midstance or any other single position) as well as complex nuances in activation among elements of a muscle and among muscles. The moments undoubtedly do contribute to muscle activation, but the complexity of the system allows for subtle changes that limit the impact of individual aspects of morphology (as suggested by, e.g., Warrener and colleagues [10] and Wall-Scheffler and Myers [23]).

Several limitations to this study are worth noting. First, we do not have whole-body energetic data (VO_2_) for these participants. Consequently, we cannot directly compare our data to that of other studies. We do not find this to be a major limitation, however, because VO_2_ would be, in this type of locomotion study, a proxy for the energy spent by the muscles. The other energetic needs of the body, such as those of maintenance or immune function, while important to the survival and reproduction of the individual, are present without regard to the whether or not the individual moves. Hence, these energetic draws are physiological “noise” if the question to be answered is about the role of locomotion in metabolic energy expenditure of muscles. Further, even if all the non-movement related oxygen consumption could be removed, the remaining oxygen consumption cannot be apportioned to individual muscles or groups of muscles, which is our focus in this research.

Second, we used a relatively simple model of the energy expenditure of muscles derived by Margaria [34] based on eccentric and concentric activations that have different energy usage. Other models exist (e.g., [35, 41]) that provide more complex thermodynamic models of muscle energy development, but their complexity requires information about muscle function that is not available for these individuals (e.g., subject specific muscle fiber length or percent fast twitch muscle fibers). Importantly, our goal was not to calculate an absolute amount of muscle energy expenditure but rather to compare relative values among individuals.

Finally, while the pelvis segment in AnyBody was allowed to scale independently in the anatomical directions during parameterization, the relative position among the points that describe the pelvis in any direction was maintained. For instance, iliac flare was a product of the superoinferior and mediolateral pelvic scale factors and was not modified independent of those factors. The shape data necessary to construct subject-specific segments was not available for the participants. Further work to explore the impact of differences in pelvic morphology on muscle metabolic energy use would be helpful.

In conclusion, our results support those of Warrener and colleagues [10] and extend their findings to slower and faster walking speeds: the mechanical conditions considered in the formulation of the OD—that is, a single, static, coronal plane snapshot of pelvic forces and moments—is too simplistic to accurately establish the energetic requirements to produce pelvic stability of the moving human body. Increasing functional pelvic width increases the maximum hip coronal plane moments as expected, but it does not follow that the energy required to stabilize the wider pelvis is also increased. The complex machine that is the human body is able to alter its movements and muscle recruitment patterns (to name but two possibilities) in subtle ways to align morphology and function [23], at least within the range of variation allowed through the evolutionary processes that produced modern humans. We should expect no less as morphology has been subject to selection in the context of function [11, 24]. Consequently, our data, and we would argue those of others who are limited to exploring the impact of modern human variation, can offer no novel explanation for why the modern female pelvis appears to be only marginally larger than the fetal head. Importantly for understanding human locomotion, behavior, and energetic adaptation, the muscle energy requirements to maintain coronal plane stability in bipedalism do not appear to be energetically expensive—or even detectably different—for modern human females compared to males. Modern human females walk as effectively as modern human males.

## Supporting information

S1 Data (XLS)

## References

[1] Heers AM. New perspectives on the ontogeny and evolution of avian locomotion. Integrative and Comparative Biology. 2016. pp. 428–441. doi: 10.1093/icb/icw065 27371381

[2] Bates KT, Mannion PD, Falkingham PL, Brusatte SL, Hutchinson JR, Otero A, et al. Temporal and phylogenetic evolution of the sauropod dinosaur body plan. R Soc Open Sci. 2016;3. doi: 10.1098/rsos.150636 27069652 PMC4821263

[3] Gembella S, Konstantinidis P, Donley JM, Sepulveda C, Shadwick RE. Evolution of High-Performance Swimming in Sharks: Transformations of the Musculotendinous system From Subcarangiform to Thunniform Swimmers. J Morphol. 2006;267: 477–493. doi: 10.1002/jmor.10412 16429422

[4] Vidal-Cordasco M, Mateos A, Zorrilla-Revilla G, Prado-Nóvoa O, Rodríguez J. Energetic cost of walking in fossil hominins. Am J Phys Anthropol. 2017;164: 609–622. doi: 10.1002/ajpa.23301 28832938

[5] Steudel-Numbers KL, Tilkens MJ. The effect of lower limb length on the energetic cost of locomotion: implications for fossil hominins. J Hum Evol. 2004;47: 95–109. doi: 10.1016/j.jhevol.2004.06.002 15288526

[6] Taylor CR, Heglund NC, Maloiy GMO. Energetics and mechanics of terrestrial locomotion I. Metabolic energy consumption as a function of speed and body size in birds and mammals. J Exp Biol. 1982; 1–22. 10.1242/jeb.97.1.1 7086334

[7] Looney DP, Potter AW, Pryor JL, Bremner PE, Chalmers CR, McClung HL, et al. Metabolic Costs of Standing and Walking in Healthy Military-Age Adults: A Meta-regression. Med Sci Sports Exerc. 2019;51: 346–351. doi: 10.1249/MSS.0000000000001779 30649093

[8] Washburn SL. Tools and Human Evolution. Sci Am. 1960;203: 62–75. 13843002

[9] Rosenberg KR. The Evolution of Modern Human Childbirth. Yearb Phys Anthropol. 1992;35: 89–124. Available: http://onlinelibrary.wiley.com.offcampus.lib.washington.edu/store/10.1002/ajpa.1330350605/asset/1330350605%5C_ftp.pdf?v=1%5C&t=gz50848k%5C&s=1f74c630d909c84208bd2e26d334fcd1643931ab

[10] Warrener AG, Lewton KL, Pontzer H, Lieberman DE. A wider pelvis does not increase locomotor cost in humans, with implications for the evolution of childbirth. PLoS One. 2015;10: 1–14. doi: 10.1371/journal.pone.0118903 25760381 PMC4356512

[11] Haeusler M, Grunstra NDS, Martin RD, Krenn VA, Fornai C, Webb NM. The obstetrical dilemma hypothesis: there’s life in the old dog yet. Biol Rev. 2021;96: 2031–2057. doi: 10.1111/brv.12744 34013651 PMC8518115

[12] Fischer B, Mitteroecker P. Allometry and Sexual Dimorphism in the Human Pelvis. Anat Rec. 2017;300: 698–705. doi: 10.1002/ar.23549 28297185

[13] Lovejoy CO, Heiple KG, Burstein AH. The Gait of Australopithecus. Am J Phys Anthropol. 1973; 757–780. doi: 10.1002/ajpa.1330380315 4735528

[14] Ruff CB. Biomechanics of the hip and birth in early Homo. Am J Phys Anthropol. 1995;98: 527–574. doi: 10.1002/ajpa.1330980412 8599386

[15] Winter DA. The Biomechanics and Motor Control of Human Gait. University of Waterloo Press, Waterloo ON; 1987.

[16] Murray MP. Gait as a total pattern of movement. Am J Phys Med. 1967;46: 290–333. Available: http://eutils.ncbi.nlm.nih.gov/entrez/eutils/elink.fcgi?dbfrom=pubmed%5C&id=5336886%5C&retmode=ref%5C&cmd=prlinks 5336886

[17] Murray MP, Mollinger LA, Gardner GM, Sepic SB. Fast Walking. J Orthop Res. 1984;2: 272–280. 6491818 10.1002/jor.1100020309

[18] Hof AL, Elzinga H, Grimmius W, Halbertsma JPK. Speed dependence of averaged EMG profiles in walking. Gait Posture. 2002;16: 78–86. doi: 10.1016/s0966-6362(01)00206-5 12127190

[19] Anders C, Sander K, Layher F, Patenge S, Kinnei RW. Temporal and spatial relationship between gluteal muscle Surface EMG activity and the vertical component of the ground reaction force during walking. PLoS One. 2021;16: 1–15. doi: 10.1371/journal.pone.0251758 34038412 PMC8153502

[20] Anders C, Patenge S, Sander K, Layher F, Biedermann U, Kinne RW. Detailed spatial characterization of superficial hip muscle activation during walking: A multi-electrode surface EMG investigation of the gluteal region in healthy older adults. PLoS One. 2017;12: 1–24. doi: 10.1371/journal.pone.0178957 28582456 PMC5459501

[21] Chumanov ES, Wall-Scheffler C, Heiderscheit BC. Gender differences in walking and running on level and inclined surfaces. Clin Biomech. 2008;23: 1260–1268. doi: 10.1016/j.clinbiomech.2008.07.011 18774631

[22] Wall-Scheffler CM, Chumanov E, Steudel-Numbers K, Heiderscheit B. Electromyography activity across gait and incline: The impact of muscular activity on human morphology. Am J Phys Anthropol. 2010;143: 601–611. doi: 10.1002/ajpa.21356 20623603 PMC3011859

[23] Wall-Scheffler CM, Myers MJ. The Biomechanical and Energetic Advantages of a Mediolaterally Wide Pelvis in Women. Anat Rec. 2017;300: 764–775. doi: 10.1002/ar.23553 28297181

[24] Sylvester AD, Lautzenheiser SG, Kramer PA. Muscle forces and the demands of human walking. Biol Open. 2021;10: 1–10. doi: 10.1242/bio.058595 34279576 PMC8325943

[25] Tague RG. Do big females have big pelves? Am J Phys Anthropol. 2000;112: 377–393. doi: 10.1002/1096-8644(200007)112:3&lt;377::AID-AJPA8&gt;3.0.CO;2-O 10861354

[26] Waltenberger L, Rebay-Salisbury K, Mitteroecker P. Age dependent changes in pelvic shape during adulthood. Anthropol Anzeiger. 2022;79: 143–156. doi: 10.1127/anthranz/2021/1463 34664055

[27] Seidel GK. Hip joint center location from palpable bony landmarks—a cadaver study. J Biomech. 1995; 1–4. 7673267 10.1016/0021-9290(94)00149-x

[28] Bennett HJ, Fleenor K, Weinhandl JT. A normative database of hip and knee joint biomechanics during dynamic tasks using anatomical regression prediction methods. J Biomech. 2018;81: 122–131. doi: 10.1016/j.jbiomech.2018.10.003 30348448

[29] Gruss LT, Gruss R, Schmitt D. Pelvic Breadth and Locomotor Kinematics in Human Evolution. Anat Rec. 2017;300: 739–751. doi: 10.1002/ar.23550 28297175 PMC6560644

[30] Rak Y. Lucy’s pelvic anatomy: its role in bipedal gait. J Hum Evol. 1991; 1–8.

[31] Stansfield E, Fischer B, Grunstra NDS, Pouca MV, Mitteroecker P. The evolution of pelvic canal shape and rotational birth in humans. BMC Biol. 2021;19: 1–11. doi: 10.1186/s12915-021-01150-w 34635119 PMC8507337

[32] Stansfield E, Kumar K, Mitteroecker P, Grunstra NDS. Biomechanical trade-offs in the pelvic floor constrain the evolution of the human birth canal. Proc Natl Acad Sci U S A. 2021;118. doi: 10.1073/pnas.2022159118 33853947 PMC8072325

[33] Ruff C. Mechanical Constraints on the Hominin Pelvis and the “Obstetrical Dilemma.” Anat Rec. 2017;300: 946–955. doi: 10.1002/ar.23539 28406558

[34] Margaria R. Positive and negative work performances and their efficiencies in human locomotion. Int Zeitschrift für Angew Physiol Einschließlich Arbeitsphysiologie. 1968;25: 339–351. doi: 10.1007/BF00699624 5658204

[35] Umberger BR, Gerritsen KGM, MARTIN PE. A Model of Human Muscle Energy Expenditure. Comput Methods Biomech Biomed Engin. 2003;6: 99–111. doi: 10.1080/1025584031000091678 12745424

[36] Lund ME, Andersen MS, de Zee M, Rasmussen J. Scaling of musculoskeletal models from static and dynamic trials. Int Biomech. 2015;2: 1–11. doi: 10.1080/23335432.2014.993706

[37] Reinbolt JA, Schutte JF, Fregly BJ, Koh B Il, Haftka RT, George AD, et al. Determination of patient-specific multi-joint kinematic models through two-level optimization. J Biomech. 2005;38: 621–626. doi: 10.1016/j.jbiomech.2004.03.031 15652563

[38] Schreiber C, Moissenet F. A multimodal dataset of human gait at different walking speeds established on injury-free adult participants. Sci Data. 2019;6: 1–7. doi: 10.1038/s41597-019-0124-4 31270327 PMC6610108

[39] Sylvester AD, Lautzenheiser SG, Kramer PA. A review of musculoskeletal modelling of human locomotion. Interface Focus. 2021;11: 20200060. doi: 10.1098/rsfs.2020.0060 34938430 PMC8361578

[40] Kramer PA, Feuerriegel EM, Lautzenheiser SG, Sylvester AD. Sensitivity of musculoskeletal models to variation in muscle architecture parameters. Evol Hum Sci. 2022;4. doi: 10.1017/ehs.2022.6 PMC10426084 37588892

[41] Bhargava LJ, Pandy MG, Anderson FC. A phenomenological model for estimating metabolic energy consumption in muscle contraction. J Biomech. 2004;37: 81–88. doi: 10.1016/s0021-9290(03)00239-2 14672571

